# Evaluation of the Wells score in predicting the incidence of stroke-associated pneumonia: the REMISE study

**DOI:** 10.3389/fneur.2025.1680293

**Published:** 2026-01-08

**Authors:** Jing Yu, Yi Liu, Jin Chen, Qin Sun, Wei Zhang, Dongze Li, Yan Zhong, Qinqin Wu, Zhi Wan

**Affiliations:** 1 Department of Emergency Medicine, Rare Diseases Center, National Clinical Research Center for Geriatrics, West China Hospital and West China School of Medicine, Sichuan University, Chengdu, China; 2 College of Arts, Sichuan University, Chengdu, China; 3 Department of Cadre Health Care, West China Hospital and West China School of Medicine, Sichuan University, Chengdu, China; 4 Health Management Center, General Practice Center, West China Hospital and West China School of Medicine, Sichuan University, Chengdu, China

**Keywords:** ischemic stroke, stroke-associated pneumonia, thrombotic burden, Wells score, cohort study

## Abstract

**Background:**

Stroke-associated pneumonia (SAP) is a common complication in patients with stroke and is strongly associated with increased mortality and disability. Thrombotic burden may serve as a potential predictor of SAP. The Wells score, widely used to estimate the probability of thrombosis, offers a practical measure of thrombotic burden. This study aimed to investigate the utility of the Wells score in predicting the risk of SAP.

**Methods:**

A total of 755 adult patients diagnosed were retrospectively included. Patients were stratified based on the Wells score into three groups: low risk group (score 0), medium risk group (score 1–2), and high risk group (score ≥3). Multivariate logistic regression analysis was performed to examine the association between the Wells score and the incidence of SAP.

**Results:**

A total of 260 patients developed SAP during hospitalization. With the increasing Wells scores, the proportion of SAP showed a rising trend (the low vs. medium vs. high risk group: 16.8% vs. 39.0% vs. 58.4%, *p* < 0.001). Multivariate logistic regression analysis showed that, compared to the low risk group, patients in the medium (odds ratio [OR]: 2.48, *p* < 0.001) and high risk group (OR: 3.49, *p* < 0.001) had more increased risks of SAP, respectively. The addition of the Wells score to A^2^DS^2^ score for SAP improved area under ROC curve.

**Conclusion:**

A high thrombotic burden is commonly observed in IS patients and is associated with an increased risk of SAP. Further research is needed to clarify the underlying mechanisms.

## Introduction

Stroke remains a leading cause of death and long-term disability worldwide, with its associated medical and economic burden continuing to grow, particularly in low- and middle-income countries (1). Although early reperfusion therapies have improved neurological outcomes, post-stroke complications remain prevalent and often determine long-term prognosis. Among these, stroke-associated pneumonia (SAP) is one of the most common and serious complications, occurring in up to 38% of hospitalized stroke patients (2–4). SAP is strongly associated with increased in-hospital mortality, prolonged hospital stays, higher healthcare costs, and unfavorable functional outcomes (5). Therefore, identifying early risk factors for SAP is critical for implementing timely preventive strategies.

The development of SAP is multifactorial. In addition to clinical and radiological characteristics, sociodemographic variables and often overlooked process-of-care-related factors, such as timing of mobilization, airway management, and oral care practices, may also contribute to the occurrence of SAP (6, 7). The A^2^DS^2^ score consisted of older age, male sex, atrial fibrillation, dysphagia, and greater stroke severity (8), is currently widely used for early risk stratification of SAP. However, it primarily reflects demographic variables and neurological impairment, with limited accuracy across heterogeneous patient populations. Our previous studies have emphasized the pivotal role of thrombo-inflammation in the pathogenesis of SAP and other stroke-related complications (4, 9–12). After stroke, the stroke-induced immunosuppression syndrome compromises pathogen clearance, while ischemia and endothelial injury trigger systemic inflammatory and enhance thrombotic burden (13, 14). Elevated thrombotic burden may reflect not only a hypercoagulable state but also underlying systemic stress, immobility, and inflammatory activation in the post-stroke period (12, 13), all of which can predispose patients to infection (15, 16, 29). These findings suggest that incorporating indicators of thrombotic burden into SAP risk assessment could enhance early identification of high-risk patients.

However, the relationship between thrombotic burden and the incidence of SAP is still poorly studied. The Wells score, a guideline-recommended clinical tool for venous thrombotic burden, is widely used to assess the probability of deep vein thrombosis (DVT) and pulmonary embolism (PE) (17, 18). Notably, all variables in the Wells score are clinically assessable and require no invasive procedures or laboratory testing. A higher Wells score indicates a greater thrombotic burden and a higher risk of progression to venous thromboembolism (VTE). But, no previous studies have investigated the potential association between the Wells score and the risk of SAP. Therefore, this study aimed to investigate whether stroke patients with a high thrombotic burden, as indicated by the Wells score, face an elevated risk of developing SAP, and to assess the predictive utility of this score in the early identification of SAP.

## Materials and methods

### Study design

The study utilized data derived from the longitudinal recovery profile of ischemic stroke patients within the Retrospective Multi-center Study for Ischemic Stroke Evaluation (REMISE) to assessed the predictive validity of the Wells score for SAP occurrence. Patients enrollment for the REMISE study occurred through the Stroke Center at West China Hospital of Sichuan University. The trial was registered at the Chinese Clinical Trial Registry (www.chictr.org.cn; Identifier: ChiCTR2100052025). Conducted in accordance with the principles of the Declaration of Helsinki, the research protocol received approval from the Human Ethics Committee of West China Hospital, Sichuan University (Approval Number: 2021–1,175).

### Study population

**Figure 1:**
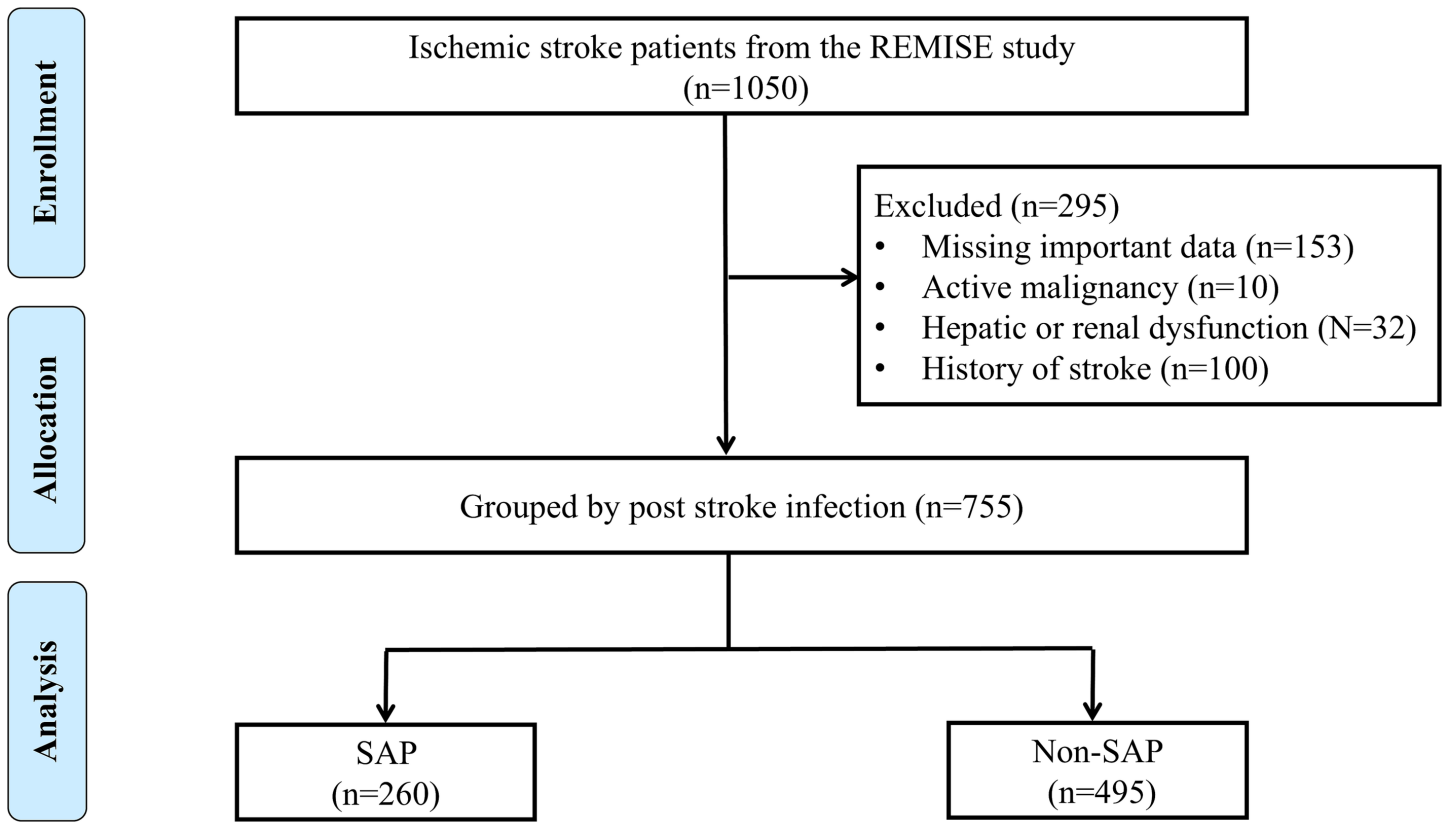
Flowchart of the study population selection.

According to the diagnostic criteria established by the American College of Cardiology/American Heart Association (ACC/AHA) (19). A total of 1,050 adult patients diagnosed with stroke were initially included. Patients were subsequently excluded for the following reasons: missing critical clinical data (*n* = 153), presence of active malignancy (*n* = 10), history of prior stroke with residual neurological deficits (*n* = 100), and significant hepatic or renal dysfunction (*n* = 32). Finally, 755 patients were included in this study (Figure 1).

### Data collection and measures

The REMISE study provided the following data categories: demographic characteristics, vital signs, medical history, medication usage, laboratory investigations, hospitalization details, and clinical outcomes. Laboratory analyses were performed using standardized systems: complete blood counts were determined with a Beckman Coulter LH750 hematology analyzer (Brea, CA), and blood biochemistry was assessed using an Abbott Architect C16000 analyzer (Dallas, TX).

The A^2^DS^2^ score (20) (range 0–10), a recognized scoring system of SAP, was calculated at admission. The stroke severity was evaluated using the NIHSS (19) (NIHSS; range 0–42) at both admission and discharge. Dysphagia was assessed using the standard bedside water-swallow test conducted by trained nurses within 24 h of admission.

### The Wells score

Two trained nurses evaluated the patients according to the Wells score (17, 18). Each clinical predictive indicator of the patient is awarded 1 point, including 9 items: (1) malignant tumors, (2) paralysis or plaster fixation of the lower extremities, (3) have been bedridden for more than 3 days recently or have undergone surgery within 28 days, (4) local tenderness in deep vein distribution, (5) edema throughout the lower extremities, (6) swelling of one side of the lower leg > 3 cm, (7) pitting edema occurs on one side, (8) history of DVT in the past, (9) lateral limb circulation with superficial veins. After adding up the scores, 0 is considered low-risk, 1 to 2 is medium-risk, and ≥3 is high-risk.

When there are differences of opinion, a higher-level physician will participate in the assessment and determination. If the patient has clinical symptoms in both lower extremities, the more severe side will be recorded. For patients with impaired consciousness or aphasia, the assessment is based on objective medical records and family reports, and is verified by a higher-level doctor.

### Outcomes

The primary outcome was the occurrence of SAP during hospitalization. According to the definition provided by the Centers for Disease Control and Prevention (CDC) (21), SAP refers to pneumonia that develops within 7 days after stroke onset in patients who did not require mechanical ventilation. The diagnosis required at least two of the following: (1) new or progressive infiltrates on chest imaging. (2) fever (>38 °C), leukocytosis (>10 × 10^9^/L) or leukopenia (<4 × 10^9^/L). (3) purulent sputum or positive sputum culture. (4) clinical signs of respiratory infection such as cough, dyspnea, or rales. SAP diagnoses were identified via electronic medical records and subsequently reviewed and verified by the study adjudication committee. Additionally, pneumonia severity was quantified via the Pneumonia Severity Index (PSI) (22), a multifactorial scoring system incorporating patient age, sex, nursing home resident, comorbidity, physical examination findings, and laboratory and radiographic results.

### Statistical analysis

Normally distributed continuous variables are presented as mean ± standard deviation (SD). Non-normally distributed continuous variables are summarized as median and interquartile range (IQR). Categorical variables are expressed as frequency counts and percentages. Between-group differences for normally distributed continuous variables were assessed using analysis of variance (ANOVA). Non-normally distributed continuous variables were compared using the Kruskal-Wallis test. Categorical variable comparisons employed the chi-square test.

Logistic regression analysis evaluated the association between the Wells score and SAP. To determine whether this association was independent of potential confounding factors, multivariable Logistic regression models were applied to calculate the odds ratios (ORs) and 95% confdence intervals (CIs), and adjusting for demographic characteristics (age, sex), behavioral factors (drinking status, smoking status), physiological parameters, laboratory values (including white blood cell count), chronic comorbidities (hypertension, diabetes, hyperlipidemia), and stroke severity (NIHSS score). The predictive capability of the Wells score for SAP was quantified using the area under the receiver operating characteristic curve (AUC).

All reported *p*-values are two-tailed. Statistical significance was defined as *p* < 0.05. Analyses were conducted using SPSS version 26.0 (IBM Corp, Armonk, NY, USA) and R software version 4.1.2 (R Foundation for Statistical Computing, Vienna, Austria).

## Results

### Baseline patient characteristics

A total of 260 patients had SAP in this study. Table 1 presents a comparison of baseline characteristics among patients with different levels of the Wells score. Patients in the higher Wells score group were significantly older, more likely to experience atrial fibrillation, dysphagia, had higher levels of white blood cell count (WBC), creatinine, blood urea nitrogen (BUN), blood glucose, D-dimer, NIHSS score, A^2^DS^2^ score, and pneumonia severity index (PSI) score, had lower levels of red blood cell count (RBC), hemoglobin, total cholesterol (TC), and albumin.

**Table 1:** Baseline characteristics according to different risk levels of the Wells score.

Variables	Low risk group (*n* = 291)	Medium risk group (*n* = 310)	High risk group (*n* = 154)	*p*-value
Age, years	62 ± 15	67 ± 13	70 ± 14	<0.001
Males, *n* (%)	193 (66.3)	198 (63.9)	84 (54.5)	<0.05
Smoking, *n* (%)	132 (45.4)	121 (39.0)	53 (34.4)	0.06
Drinking, *n* (%)	103 (35.4)	86 (27.7)	37 (24.0)	<0.05
Hypertension, *n* (%)	173 (59.5)	186 (60.0)	100 (64.9)	0.49
Diabetes, *n* (%)	69 (23.7)	81 (26.1)	38 (24.7)	0.79
Hyperlipidemia, *n* (%)	30 (10.3)	32 (10.3)	8 (5.2)	0.15
Atrial fibrillation, *n* (%)	44 (15.1)	90 (29.0)	55 (35.7)	<0.001
Dysphagia, *n* (%)	16 (5.5)	22 (7.1)	34 (22.1)	<0.001
SBP, mmHg	145 ± 24	146 ± 25	146 ± 26	0.90
DBP, mmHg	86 ± 16	87 ± 16	87 ± 15	0.79
Heart rate, beats/min	78 (70–89)	80 (67–90)	81 (69–98)	0.10
BMI, Kg/m^2^	22.1 ± 7.5	20.2 ± 8.8	18.7 ± 6.6	<0.001
Laboratory findings				
RBC, 10^12^/L	4.55 ± 0.57	4.51 ± 0.61	4.31 ± 0.74	<0.001
WBC, 10^9^/L	7.21 ± 2.51	7.89 ± 2.76	8.63 ± 3.30	<0.001
PLT, 10^9^/L	185 ± 70	180 ± 71	189 ± 79	0.45
Hemoglobin, g/L	137.95 ± 17.31	136.66 ± 17.58	129.83 ± 21.85	<0.001
Creatinine, μmol/L	71.5 (60.8–85.0)	74.0 (64.0–85.2)	76.0 (64.0–95.0)	<0.05
BUN, mmol/L	5.5 (4.38–6.73)	5.7 (4.5–7.1)	6.1 (4.5–8.3)	<0.05
Blood glucose, mmol/L	7.17 ± 2.84	7.98 ± 3.10	8.28 ± 3.63	<0.001
LDL, mmol/L	2.36 (1.81–3.12)	2.53 (1.94–3.16)	2.34 (1.83–2.82)	0.08
HDL, mmol/L	1.27 ± 0.39	1.26 ± 0.40	1.19 ± 0.39	0.14
TC, mmol/L	4.75 ± 1.31	4.41 ± 1.23	4.27 ± 1.81	<0.001
TG, mmol/L	1.3 (0.9–2.0)	1.2 (0.9–2.0)	1.1 (0.9–1.8)	0.77
Albumin, g/L	42.83 ± 3.33	41.41 ± 4.02	39.44 ± 4.14	<0.001
D-dimer, mg/L	0.37 (0.21–0.91)	0.82 (0.35–1.73)	1.54 (0.61–4.64)	<0.001
Risk scores				
NIHSS score	2 (1–5)	11 (5–16)	13 (8–18)	<0.001
A^2^DS^2^ score	3 (1–4)	4 (3–6)	5 (4–7)	<0.001

SBP, systolic blood pressure; DBP, diastolic blood pressure; BMI, body mass index; RBC, red blood cell count; WBC, white blood cell count; PLT, platelet count; BUN, blood urea nitrogen; LDL, low-density lipoprotein; HDL, high-density lipoprotein; TC, Total cholesterol; TG, triglycerides; NIHSS, National Institute of Health Stroke Scale.

### Relationship between the Wells score and stroke-associated pneumonia

The proportion of SAP was significantly higher in patients with a high Wells score compared to those with a low Wells score (the low vs. medium vs. high risk group: 16.8% vs. 39.0% vs. 58.4%, *p* < 0.001). Similarly, the risk of SPA increased with the increasing Wells score. In the multivariable logistic regression model, adjusted for demographic characteristics, laboratory parameters, and chronic comorbidities, demonstrated that the Wells score remained an independent predictor of SAP (Table 2). Compared to the low risk group, patients in the medium risk group had a 2.48-fold increased risk of developing SAP (OR: 2.48; 95% CI: 1.42–4.33; *p* < 0.001), while those in the high risk group exhibited a 3.49-fold higher risk (OR: 3.49; 95% CI: 1.81–6.72; *p* < 0.001). Multivariate logistic regression analysis revealed a 2.14-fold increase in SAP risk per one unit increase in the Wells score (OR: 2.14; 95% CI: 1.68–2.73; *p* < 0.001).

**Table 2:** Logistic regression models for the relationship between the Wells score and the risk of stroke-associated pneumonia.

Variables	Model 1		Model 2		Model 3	
	OR (95% CI)	*p*-value	OR (95% CI)	*p*-value	OR (95% CI)	*p*-value
Wells score (category)		<0.001		<0.001		<0.001
Low	1		1		1	
Medium	2.64 (1.72–4.04)	<0.001	2.45 (1.58–3.81)	<0.001	2.48 (1.42–4.33)	0.01
High	5.45 (3.29–9.06)	<0.001	5.26 (3.11–8.88)	<0.001	3.49 (1.81–6.72)	<0.001
Wells score (per 1 unit increase)	2.17 (1.72–2.76)	<0.001	2.12 (1.66–2.71)	<0.001	2.14 (1.68–2.73)	<0.001

Model 1 adjusted for age, sex, smoking, drinking, body mass index, dysphagia.

Model 2 adjusted for age, sex, smoking, drinking, body mass index, dysphagia, hypertension, diabetes, hyperlipidemia, atrial fibrillation.

Model 3 adjusted for age, sex, smoking, drinking, body mass index, dysphagia, hypertension, diabetes, hyperlipidemia, atrial fibrillation, white blood cell count, platelet count, D-dimer.

OR, odds ratio; CI, confidence interval.

### Predictive value of the Wells score and stroke-associated pneumonia

**Figure 2:**
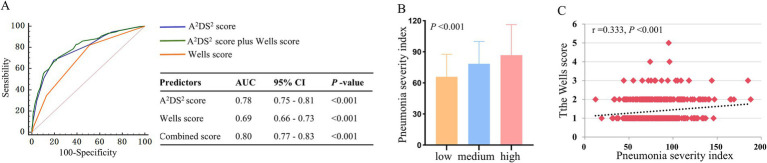
**(A)** Receiver operating characteristic curve analysis of the Wells score, A^2^DS^2^ score, and the combined score for stroke-associated pneumonia. **(B)** The pneumonia severity index among patients with different risk levels of the Wells score. **(C)** The Spearman’s correlation analysis between the Wells score and pneumonia severity index.

Receiver operating characteristic (ROC) curve analysis demonstrated that the Wells score and A^2^DS^2^ score for SAP showed AUC is 0.69 (95% CI: 0.66–0.73; *p* < 0.001) and 0.78 (95% CI: 0.75–0.81; *p* < 0.001), respectively. Combining the Wells score with the A^2^DS^2^ score significantly enhanced predictive performance (AUC: 0.80; 95% CI: 0.77–0.83; *p* < 0.001), which was higher than either score alone (*p* < 0.001 for all comparisons; Figure 2A).

The diagnostic test characteristics (Table 3) showed that the Wells score threshold of ≤1 provided optimal sensitivity (81.15%) and negative predictive value (NPV, 83.16%) for SAP detection. Conversely, a threshold of ≥3 showed the highest specificity (98.99%) for identifying high-risk patients.

**Table 3:** The predictive value of Wells score for stroke-associated pneumonia.

Fall risk score	Sensitivity, %	Specificity, %	Accuracy, %	PPV, %	NPV, %
≥0	100	0	34.44	34.44	-
≥1	81.15	48.89	60.00	45.47	83.16
≥2	34.62	87.07	69.01	58.44	71.71
≥3	4.23	98.99	66.36	68.75	66.31

PPV, positive predictive value; NPV, negative predictive value.

### Association of the Wells score with the severity of pneumonia

Patients with higher levels of the Wells score had more elevated pneumonia severity index (low vs. medium vs. high risk group: 65.82 ± 21.72 vs. 78.46 ± 21.52, vs. 86.71 ± 29.45, *p* < 0.001, Figure 2B). Spearman’s correlation analysis indicated that there was a positive correlation between the Wells score and pneumonia severity index (*r* = 0.333, *p* < 0.001, Figure 2C).

### Subgroup analysis

Consistency of the Wells score’s association with SAP was evaluated across prespecified subgroups stratified by age, gender, hypertension status, alcohol consumption, smoking history, diabetes, hyperlipidemia, atrial fibrillation, WBC, and NIHSS score (Table 4). The association between elevated Wells score category and increased SAP risk remained statistically significant and consistent across in subgroups.

**Table 4:** Subgroup analysis of the association between the Wells score with stroke-associated pneumonia.

Variable	Medium risk vs. Low risk			High risk vs. Low risk		*p* for interaction
	OR (95% CI)	*p*-value		OR (95% CI)	*p*-value	
Age, years						
<65	3.17 (1.74–5.77)	<0.001		5.18 (2.50–10.72)	<0.001	0.53
≥65	2.71 (1.63–4.51)	<0.001		6.58 (3.67–11.81)	<0.001	
Sex						
Male	3.27 (2.04–5.26)	<0.001		5.53 (3.12–9.81)	<0.001	0.21
Female	2.97 (1.56–5.67)	<0.001		9.13 (4.45–18.74)	<0.001	
Drinking						
No	3.60 (2.25–5.78)	<0.001		7.31 (4.28–12.49)	<0.001	0.91
Yes	2.37 (1.22–4.61)	0.011		6.48 (2.85–14.78)	<0.001	
Smoking						
No	3.29 (1.99–5.42)	<0.001		7.14 (4.03–12.62)	<0.001	0.97
Yes	2.91 (1.60–5.29)	<0.001		6.38 (3.13–13.05)	<0.001	
Hypertension						
No	3.48 (1.94–6.23)	<0.001		6.86 (3.35–14.04)	<0.001	0.85
Yes	2.97 (1.79–4.95)	<0.001		7.17 (4.05–12.68)	<0.001	
Diabetes						
No	3.30 (2.12–5.12)	<0.001		7.61 (4.56–12.71)	<0.001	0.49
Yes	2.79 (1.30–6.03) -	0.009		5.28 (2.17–12.86)	<0.001	
Hyperlipidemia						
No	3.53 (2.36–5.28)	<0.001		6.68 (4.21–10.60)	<0.001	0.35
Yes	0.93 (0.24–3.59)	0.911		15.00 (2.32–96.96)	0.004	
Atrial fibrillation						
No	2.79 (1.73–4.50)	<0.001		6.51 (3.75–11.30)	<0.001	0.40
Yes	2.50 (1.19–5.22)	0.015		4.23 (1.80–9.94)	<0.001	
WBC, 10^9^/L						
≤7	4.78 (2.60–8.80)	<0.001		7.05 (3.35–14.80)	<0.001	0.74
>7	2.01 (1.21–3.34)	0.007		4.99 (2.80–8.89)	<0.001	
NIHSS score						
≤7	1.64 (0.81–3.31)	0.17		1.18 (0.37–3.79)	0.78	0.54
>7	1.04 (0.51–2.12)	0.92		1.78 (0.82–3.85)	0.14	

WBC, white blood cell count; NIHSS National Institute of Health Stroke Scale.

## Discussion

This study confirmed that a high thrombotic burden is prevalent among patients with stroke and is independently associated with an increased risk of SAP. After adjusting for potential confounders, the higher Wells score remained a significant independent predictor of SAP. This association was consistent across different subgroups. The thrombotic burden provides additional prognostic information beyond traditional risk factors. Although the Wells score showed statistically significant discrimination for SAP, its AUC of 0.69 indicates only moderate predictive performance. When combined with the A^2^DS^2^ score, however, predictive accuracy improved. These findings suggest that the Wells score may serve as a novel and independent clinical tool for early SAP risk stratification. This expands the clinical applicability of the Wells score beyond its traditional role in VTE assessment and emphasizing the potential importance of antithrombotic management in improving outcomes for stroke patients.

Multiple interrelated mechanisms, including infection, dysphagia, stroke-induced immunosuppression, and impaired consciousness, collectively contribute to the development of SAP (13). Meanwhile systemic inflammation, endothelial dysfunction, and metabolic disturbances also play important roles in SAP development. Previous studies have shown that acute infections, including pneumonia, significantly increase the risk of VTE in both community-dwelling populations and patients with COVID-19 (23–25, 30). Coagulation biomarkers such as prothrombin time and activated partial thromboplastin time have also been linked to stroke risk (26, 29). In our study, patients with higher Wells scores exhibited elevated white blood cell counts, greater stroke severity, and increased PSI scores, which further support the hypothesis that increased thrombotic burden enhances susceptibility to SAP. The prevalence of smoking in our cohort was relatively high, which may reflect regional and demographic characteristics of stroke populations. Smoking is a well-established pro-thrombotic and pro-inflammatory factor that contributes to endothelial injury, platelet activation, and hypercoagulability, thereby potentially amplifying the association between thrombotic burden and SAP risk. In contrast, among patients with mild neurological deficits (NIHSS ≤7), the association between the Wells score and SAP was not statistically significant. This may be explained by their better mobility, lower systemic inflammation, and shorter hospital stays, which reduce thrombotic burden and SAP risk.

The predictive value of thrombotic burden for SAP may lie in its ability to capture overlapping thrombo-inflammatory mechanisms. Reduced mobility following stroke leads to venous stasis and systemic inflammation, which increase thrombotic load and simultaneously elevate infection risk (27). Recent studies have identified neutrophil extracellular traps (NETs), hallmarks of immunothrombosis, in the circulation of stroke patients. NETs not only promote fibrin-rich clot formation but also impair vascular integrity and weaken immune defense, thus establishing a biological link between thrombosis and infection (14). Additionally, our findings showed that malnutrition may mediate this association. Patients in the high Wells score group had significantly lower serum albumin and total cholesterol levels in this study. These findings are consistent with previous studies showing that hypoalbuminemia exacerbates endothelial dysfunction, impairs the synthesis of anticoagulant proteins, and promotes catabolism (16). Thereby, the thrombotic load after stroke may increase the SAP susceptibility. The Wells score, as a simple scoring system for thrombotic load, was identified to has predictive value for SAP in this present study.

Moreover, combining the Wells score with the A^2^DS^2^ score improved early discrimination of SAP risk in stroke patients. While the A^2^DS^2^ score emphasizes neurologic deficits and dysphagia, the Wells score reflects the systemic thrombotic burden. Early use of the Wells score may address limitations of conventional models such as A^2^DS^2^, particularly in patients with a hypercoagulable state. Unlike many thrombotic risk assessment models, the Wells score offers distinct clinical advantages: it is simple to use, reproducible, and does not require laboratory testing. These characteristics align with emerging concepts that prognostic models integrating inflammation and thrombosis status offer enhanced predictive performance for post-stroke complications (28). Thus, early risk stratification based on the Wells score may guide interventions for SAP, such as intensive antithrombotic drugs.

However, several limitations should be acknowledged. First, although the sample size was adequate, this was a multicenter retrospective study conducted in emergency departments of general hospitals, which may introduce selection bias and limit causal inference. Second, there was no dynamic observation of biomarkers associated with thrombotic load, limiting mechanistic insights. And patients with late-onset pneumonia or those requiring mechanical ventilation were excluded, which may affect the generalizability of the findings. The Wells score was assessed only at admission, without follow-up evaluation, which may influence the consistency of the association over time. Additionally, the Wells score was not directly compared with other thrombosis risk assessment tools such as the Padua scores or Caprini scores in this study. Future studies are needed to determine the superior predictive for SAP.

## Conclusion

This multicenter retrospective study demonstrates that the Wells score can independently predict the risk of SAP in patients with stroke. When combined with the A^2^DS^2^ score, it significantly enhances predictive accuracy. Therefore, thrombus burden may serve as a target for preventing SAP. Future studies should focus on the benefits of thrombus intervention for SAP and explore mechanisms to optimize prediction and intervention strategies.

## Data Availability

The raw data supporting the conclusions of this article will be made available by the authors, without undue reservation.

## Glossary

BMI: body mass index
BUN: blood urea nitrogen
CI: confidence interval
DVT: deep vein thrombosis
DBP: diastolic blood pressure
HDL: high-density lipoprotein
NETs: neutrophil extracellular traps
LDL: low-density lipoprotein
NIHSS: National Institute of Health Stroke Scale
NPV: negative predictive value
OR: odds ratio
PPV: positive predictive value
PSI: pneumonia severity index
PE: pulmonary embolism
PLT: platelet count
REMISE: Retrospective Multi-center Study for Ischemic Stroke Evaluation
ROC: Receiver operating characteristic
RBC: red blood cell count
SAP: Stroke-associated pneumonia
SBP: systolic blood pressure
TC: Total cholesterol
TG: triglycerides
VTE: venous thromboembolism
WBC: white blood cell count
